# Prof. Milton G. Potter, M. D.

**Published:** 1878-03

**Authors:** 


### OBITUARIES.

Died, at his home in this city on Monday, January 28th, 1878, Professor Milton G. Potter, M. D., in the thirty-fifth year of his age.

Tuesday evening, January 29th 1878 a largely attended meeting of the Erie County Medical Society was held at the College building, to take action in reference to the death of Dr. Milton Grosvenor Potter, the President, Dr. E. Storck in the chair, and Dr. D. W. Harrington at his post as Secretary.

Upon calling the meeting to order, the President, Dr. E. Storck, said that in paying tribute to the memory of Dr. Potter, he was reminded that the deceased was the representative of the younger practitioners in Buffalo. Though a young physician, he had attained a standing of which he could well feel proud. He was an ardent student as well as a conscientious practitioner and able teacher, and his memory is dear to all, "the life of the dead is in the memory of the living."

Dr. Storck was followed by Prof. White, who said that he regarded Dr. Potter as his child. His professional attainments and success as a practitioner were extraordinary. As a teacher he was brilliant, and taught more effectually and thoroughly than any of his predecessors in the faculty. He siezed all the salient points, dwelt upon and reiterated them until they were fairly imbedded in the memory of those he taughl. By his class he was always loved, and his future was foreshadowed in the oration delivered by him at the time he graduated. He sacrificed him self to what he thought to be a duty. To the speaker he was endeared by many ties. They had been constant companions for several years, traveling in various parts of the States of New York, Michigan and Rhode Island; and Prof. White knew him but to love him. He was eminently qualified for a surgeon; cool, ready, clear-headed, sagacious in diagnosis, prompt in execution. The speaker never saw a man upon whom more reliance could be placed in important and difficult operations. His duties as Dean of the Faculty added to his labors as Professor of Anatomy, and in the spring of 1875, at the conclusion of the course, he was overwhelmed with nervous prostration. Disregarding the admonitions of himself and others, after a brief interval he continued his labors and delivered the course for 1875 and 1876 with difficulty, in consequence of great nervous exhaustion and physical disability.

After mature consultation it was deemed important that he should rest and try the effect of change of climate. In the latter part of last summer he returned to Buffalo, resumed his professional pursuits, and in the fall commenced the delivery of his lectures with more than his ordinary zeal and thoroughness. Heroically he struggled on, never flagging in his labors until December, when he was compelled reluctantly to yield to the overwhelming prostration incident to the progress of his disease.

From that time he was a great sufferer notwithstanding every effort which his physician, Dr. Rochester, aided by the speaker and his other colleagues put forth, he rapidly failed in strength until the morning of January 28th, when death closed the scene. With a comfortable, religious and holy hope as to the future, and without murmuring that his earthly career was thus prematurely ended, he passed peacefully to rest.

Remarks were then made by Prof. Rochester, Dr. Abbott, Dr. Elwood, Dr. J. S. Smith, Dr. Phelps and Dr. O'Brien. At the conclusion of Dr. O'Brien's remarks, resolutions were adopted that the society attend the funeral of Dr. Potter in a body and that Dr. E. N. Brush be requested to prepare an eulogium upon Dr. Potter, to be read at some future meeting.

